# Sex Difference in the Associations among Obesity-Related Indices with Hyperuricemia in a Large Taiwanese Population Study

**DOI:** 10.3390/nu15153419

**Published:** 2023-08-01

**Authors:** Shih-Yao Su, Tsung-Han Lin, Yi-Hsueh Liu, Pei-Yu Wu, Jiun-Chi Huang, Ho-Ming Su, Szu-Chia Chen

**Affiliations:** 1 Department of Medicine, Kaohsiung Medical University, Kaohsiung 807, Taiwan; ionsu93@gmail.com; 2 Department of Internal Medicine, Kaohsiung Municipal Ta-Tung Hospital, Kaohsiung Medical University Hospital, Kaohsiung Medical University, Kaohsiung 807, Taiwan; moresque612@yahoo.com; 3 Division of Cardiology, Department of Internal Medicine, Kaohsiung Medical University Hospital, Kaohsiung Medical University, Kaohsiung 807, Taiwan; liuboy17@gmail.com (Y.-H.L.); cobeshm@seed.net.tw (H.-M.S.); 4 Department of Internal Medicine, Kaohsiung Municipal Siaogang Hospital, Kaohsiung Medical University Hospital, Kaohsiung Medical University, Kaohsiung 812, Taiwan; wpuw17@gmail.com (P.-Y.W.); karajan77@gmail.com (J.-C.H.); 5 Division of Nephrology, Department of Internal Medicine, Kaohsiung Medical University Hospital, Kaohsiung Medical University, Kaohsiung 807, Taiwan; 6 Faculty of Medicine, College of Medicine, Kaohsiung Medical University, Kaohsiung 807, Taiwan; 7 Research Center for Precision Environmental Medicine, Kaohsiung Medical University, Kaohsiung 807, Taiwan

**Keywords:** obesity-related indices, waist-to-height ratio, waist–hip ratio, lipid accumulation product, hyperuricemia, Taiwan Biobank

## Abstract

Hyperuricemia has been linked with the development of diabetes, gout, kidney, and cardiovascular diseases. Although obesity is associated with hyperuricemia, data on sex differences in this association are scarce. Therefore, this study was conducted to explore sex differences in the correlations among various indices of obesity with hyperuricemia in Taiwan. Data were obtained from the Taiwan Biobank and included 122,067 participants. After excluding 179 participants with missing data, the remaining 121,888 participants (men: 43,790; women: 78,098) were enrolled. The prevalence rates of hyperuricemia (defined as serum uric acid >7.0/6.0 mg/dL in men/women) were 29.8% and 13.6%, respectively (*p* < 0.001). Multivariable analysis revealed high values of body shape index (ABSI), waist-to-height ratio (WHtR), waist–hip ratio (WHR), lipid accumulation product (LAP), conicity index (CI), visceral adiposity index (VAI), body adiposity index (BAI), abdominal volume index (AVI), body mass index (BMI), and body roundness index (BRI) were significantly associated with hyperuricemia in both the male and female participants (all *p* < 0.001). The interactions between sex and all 10 of these indices were significant (all *p* < 0.001) for hyperuricemia. In men, LAP had the highest area under the curve (0.669), followed by BMI (0.655), VAI (0.645), AVI (0.642), BRI (0.640), WHtR (0.633), BAI (0.605), WHR (0.599), CI (0.574), and ABSI (0.510). In women, LAP also had the highest area under the curve (0.754), followed by BMI (0.728), VAI (0.724), WHtR (0.721), BRI (0.720), AVI (0.713), WHR (0.676), BAI (0.673), CI (0.626), and ABSI (0.544). In conclusion, obesity-related indices were associated with hyperuricemia in this large Taiwanese study, and sex differences were found in these associations, with stronger associations in women than in men.

## 1. Introduction

Hyperuricemia is caused by an excess of uric acid (UA) as a result of disorders such as abnormal purine metabolism and reduced UA excretion [1,2]. Previous epidemiological studies have reported different prevalence rates between different areas. For example, the reported prevalence of hyperuricemia in the US is approximately 20% in both women and men [3] compared to 8.4–25% in Chinese adults [4]. In addition, the reported prevalence rates in Taiwan are 9.7% and 22.0% in women and men, respectively [5]. Hyperuricemia is a growing health problem worldwide, and measures to address this issue are urgently required.

Purine is metabolized in humans through the catalyzation by xanthine oxidase of hypoxanthine to xanthine and then UA [6]. Moreover, the subsequent generation of reactive oxygen species has been associated with the development of metabolic disorders [6]. These findings suggest the role of hyperuricemia in various systemic diseases. The development of hyperuricemia has also been linked to poor renal function, obesity, older age, and male sex [7,8,9], and hyperuricemia has been associated with higher risks of gout [7] and cardiovascular disorders, including high left atrial diameter and low left ventricular ejection fraction [10]. Hyperuricemia has also been associated with chronic kidney disease, thyroid dysfunction, metabolic syndrome, hypertension, and dyslipidemia [7,9,10,11,12]. Hence, identifying other factors that may be associated with hyperuricemia is important to address the increased burden on healthcare systems and develop new treatments to improve patient care.

Obesity is defined as excess adipose tissue accumulation, and it is also a major health issue [13]. Approximately 2 billion adults were estimated to be overweight or obese in 2015, representing approximately 39% of the global population [14]. Moreover, there was an almost 50% increase in the age-standardized prevalence of overweight from 26.5% in 1980 to 39.0% in 2015 [14]. Obesity is related to numerous metabolic syndromes, including insulin resistance, dyslipidemia, and hyperuricemia, and also increased rates of cardiovascular morbidity and mortality [15,16]. Obesity can be evaluated using a variety of indices, including body mass index (BMI) [17], waist-to-hip ratio (WHR), waist-to-height ratio (WHtR), lipid accumulation product (LAP), body roundness index (BRI) [18], conicity index (CI) [19], visceral adiposity index (VAI) [20], body adiposity index (BAI) [21], abdominal volume index (AVI) [22], and body shape index (ABSI) [23]. Quantifying obesity using these indices can help to evaluate their relationships with other health problems, and we previously identified associations between these obesity indices with fatty liver [24], albuminuria, advanced kidney disease [25], lung function [26], osteoporosis [27], hypertension [28], peripheral artery disease [29], and dementia [30].

Of the known risk factors for hyperuricemia, including older age and obesity/overweight [31], obesity has been independently associated with its development [32,33,34]. Moreover, serum UA, which is an indicator of hyperuricemia, has been positively related to several obesity-related indices, including BMI [35,36,37,38]. Even though relationships between obesity-related indices and hyperuricemia are well documented, the effects of sex differences on this relationship remain unclear. Therefore, we conducted this study to investigate sex differences in the associations among obesity-related indices with hyperuricemia in a large Taiwanese population.

## 2. Materials and Methods

### 2.1. The Taiwan Biobank

The Taiwan Biobank is a large research initiative launched in 2008 to record health-related data from community-dwelling Taiwanese participants, including genetic information, lifestyle habits, and health records [39,40]. All participants in the Taiwan Biobank are asked to give written informed consent during an interview before biological samples are collected and physical examinations are conducted.

### 2.2. Study Participants

**Figure 1:**
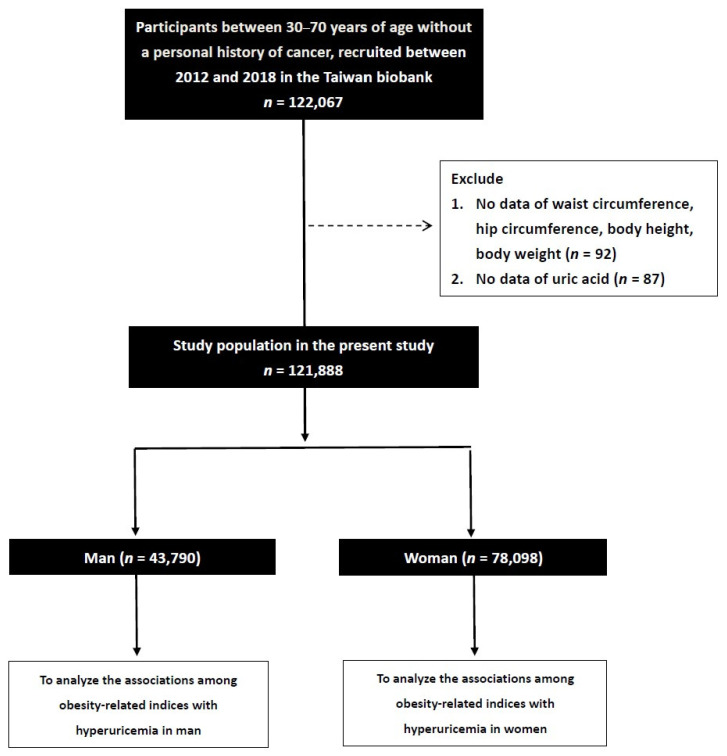
Flowchart of study population. This study was conducted to explore sex differences in the correlations among various indices of obesity with hyperuricemia.

Of 122,067 subjects collected from the Taiwan Biobank, we excluded those who lacked information on UA (*n* = 87) and those without information on body weight and height (BW and BH) and hip and waist circumference (HC and WC) (*n =* 92). We then stratified the remaining 121,888 participants (men: 43,790; women: 78,098; mean age 49.9 ± 10.9 years) according to whether or not they had hyperuricemia. In this study, we used a definition of hyperuricemia of UA serum concentration greater than 7.0 and 6.0 mg/dL in the male and female enrollees, respectively [41] (Figure 1).

### 2.3. Collection of Study Variables

Data obtained from the Taiwan Biobank included BW, BH, systolic/diastolic blood pressure (BP), HC, and WC. Data obtained when the enrollees were interviewed included medical history (hypertension and diabetes mellitus), smoking status, age, and sex. 

Other data, including high-density lipoprotein cholesterol (HDL-c), low-density lipoprotein cholesterol (LDL-c), total cholesterol, triglycerides (TGs), hemoglobin, fasting glucose, UA, and estimated glomerular filtration rate (eGFR; calculated as described previously [42]), were also obtained.

Three BP measurements were recorded after 1–2-min breaks using an automated sphygmomanometer with the patients asked to not smoke, exercise, or ingest caffeine for at least 30 min beforehand. Average BP values were used in the analysis.

### 2.4. Obesity-Related Indices Calculations

1. Body mass index:
  BMI = BW (kg)/BH^2^ (m)
2. Waist–hip ratio:
  WHR = WC (cm)/HC (cm)
3. Waist-to-height ratio:
  WHtR = WC (cm)/BH (cm)
4. Body roundness index [18]:
  $$\mathrm{BRI}=364.2-365.5\times \sqrt{1-{\left(\frac{\frac{{WC}_{\left(m\right)}}{2\pi }}{0.5\times {BH}_{\left(m\right)}}\right)}^{2}\;}$$
5. Conicity index [19]:
  $$\mathrm{CI}=\frac{{WC}_{\left(m\right)}}{0.109\times \sqrt{\frac{{BW}_{\left(kg\right)}}{{BH}_{\left(m\right)}}}\;}$$
6. Body adiposity index [21]:
  $$\mathrm{BAI}=\frac{{HC}_{\left(cm\right)}}{{{BH}_{\left(m\right)}}^{3/2}}-18$$
7. Abdominal volume index (AVI) [22]:
  $$\mathrm{AVI}=\frac{2\times {\left({WC}_{\left(cm\right)}\right)}^{2}+0.7\times {\left({WC}_{\left(cm\right)}-{HC}_{\left(cm\right)}\right)}^{2}}{1000}\;$$
8. A body shape index (ABSI) [23]:
  ABSI = WC (m)/[BMI^2/3^(kg/m^2^) × BH^1/2^(m)]
9. Lipid accumulation product [17]:
  $$\begin{array}{c} \mathrm{LAP}=\left({WC}_{\left(cm\right)}-65\right)\times {TG}_{\left(mmol/L\right)}\;\mathrm{in males},\;\mathrm{and}\\ \mathrm{LAP}=\left({WC}_{\left(cm\right)}-58\right)\times {TG}_{\left(mmol/L\right)}\;\mathrm{in females} \end{array}$$
10. Visceral adiposity index:
  $$\begin{array}{c} \mathrm{VAI}=\left(\frac{{WC}_{\left(cm\right)}}{39.68+\left(1.88\times BMI\right)}\right)\times \left(\frac{{TG}_{\left(mmol/L\right)}}{1.03}\right)\times \left(\frac{1.31}{{HDL-c}_{(mmol/L)}}\right)\;\mathrm{in males},\;\mathrm{and}\\ \mathrm{VAI}=\left(\frac{{WC}_{\left(cm\right)}}{36.58+\left(1.89\times BMI\right)}\right)\times \left(\frac{{TG}_{\left(mmol/L\right)}}{0.81}\right)\times \left(\frac{1.52}{{HDL-c}_{(mmol/L)}}\right)\;\mathrm{in females}. \end{array}$$

### 2.5. Ethics Statement

All Taiwan Biobank enrollees gave informed consent in written form. Ethical approval for the Taiwan Biobank was granted by the IRB on Biomedical Science Research, Academia Sinica, Taiwan, and the Ethics and Governance Council of the Taiwan Biobank. The Institutional Review Board of Kaohsiung Medical University Hospital approved our study (KMUHIRB-E(I)-20210058 and 2021/4/8 approval), which was performed according to the Helsinki Declaration.

### 2.6. Statistical Analysis

Data are reported in percentages or represented as the mean ± standard deviation. Independent *t*-tests were used for comparing continuous variables, and the chi-square test for categorical variables. The normal distribution of variables was evaluated with the Kolmogorov–Smirnov test. Homogeneity of variance was tested with Levene’s test. Levene’s test was used to assess the equality of variances and an independent sample *t*-test. A two-tailed *p*-value less than 0.05 was considered statistically significant. Factors associated with hyperuricemia were identified using multivariable logistic regression analyses. An interaction *p* in logistic analysis: Model disease (y) = x1 + x2 + x1 × x2 + covariates. x1 × x2 was the interaction term, where y = hyperuricemia; x1 = sex; x2 = each obesity-related index; covariates = age, diabetes mellitus, hypertension, smoking history, systolic and diastolic BPs, hemoglobin, TGs, total cholesterol, HDL-c, LDL-c, and eGFR. Receiver operating characteristic (ROC) curve analysis was used to evaluate the performance of each obesity-related index to predict the incidence of hyperuricemia. The predictive ability of each obesity-related index was evaluated according to the area under ROC curve (AUC). The *p*-values <0.05 were considered significant. All statistical calculations were performed with SPSS version 19 (IBM Inc., Armonk, NY, USA).

## 3. Results

In the 121,888 enrolled participants, the prevalence of hyperuricemia was 29.8% in men and 13.6% in women (*p* < 0.001).

### 3.1. Differences between the Male and Female Participants with and without Hyperuricemia in Clinical Characteristics

Comparisons of clinical characteristics between the male and female participants with and without hyperuricemia groups are shown in Table 1. The male with hyperuricemia group was younger, had a lower prevalence rate of diabetes mellitus, higher prevalence rates of hypertension and smoking history, higher systolic and diastolic BPs, higher BH, BW, WC, HC, UA, hemoglobin, TGs, total cholesterol, and LDL-c, and lower fasting glucose and HDL-c compared with the male without hyperuricemia group. Moreover, the male with hyperuricemia group had greater values of all 10 obesity-related indices (all *p* < 0.001).

In addition, the female with hyperuricemia group was older, had a higher prevalence of diabetes mellitus and hypertension, higher systolic and diastolic BP, higher BH, BW, WC, HC, UA, fasting glucose, hemoglobin, TGs, total cholesterol, LDL-c, and eGFR, and lower HDL-c compared with women who did not have hyperuricemia. Furthermore, the women who had hyperuricemia also had greater values of all 10 obesity-related indices (all *p* < 0.001).

### 3.2. Sex Differences in the Associations between the Obesity-Related Indexes and Hyperuricemia 

The results of the multivariable logistic regression analysis for sex differences in the associations between the obesity-related indexes and hyperuricemia are shown in Table 2. Three models were used in the analysis.

1. For BMI, WHR, WHtR, BRI, CI, BAI, AVI, and ABSI: ^a^ adjustments for age, diabetes mellitus, hypertension, smoking status, systolic and diastolic BP, hemoglobin, TGs, total cholesterol, HDL-c, LDL-c, and eGFR.
2. For LAP: ^b^ adjustments as for model 1 but without TGs.
3. For VAI: ^c^ adjustments as for model 2 but without HDL-c.

The results showed that high values of BMI (per 1 kg/m^2^; odds ratio (OR) = 1.121; *p* < 0.001), WHR (per 0.01; OR = 1.058; *p* < 0.001), WHtR (per 0.01; OR = 1.077; *p* < 0.001), BRI (per 1; OR = 1.241; *p* < 0.001), CI (per 1; OR = 1.460; *p* < 0.001, BAI (per 1; OR = 1.095; *p* < 0.001), AVI (per 1; OR = 1.119; *p* < 0.001), ABSI (per 0.01; OR = 1.260; *p* < 0.001), LAP (per 1; OR = 1.010; *p* < 0.001), and VAI (per 1; OR = 1.238; *p* < 0.001) were significantly associated with hyperuricemia in men. Similarly, high values of BMI (per 1 kg/m^2^; OR = 1.163; *p* < 0.001), WHR (per 0.01; OR = 1.054; *p* < 0.001), WHtR (per 0.01; OR = 1.090; *p* < 0.001), BRI (per 1; OR = 1.296; *p* < 0.001), CI (per 0.1; OR = 1.340; *p* < 0.001), BAI (per 1; OR = 1.113; *p* < 0.001), AVI (per 1; OR = 1.162; *p* < 0.001), ABSI (per 0.01; OR = 1.088; *p* < 0.001), LAP (per 1; OR = 1.018; *p* < 0.001), and VAI (per 1; OR = 1.339; *p* < 0.001) were significantly associated with hyperuricemia in women.

### 3.3. Interactions between Sex and the Obesity-Related Indexes on Hyperuricemia

Significant interactions were found between sex and all 10 obesity-related indices (all *p* < 0.001) on hyperuricemia (Table 2).

### 3.4. Performance and Predictive Ability of the Obesity-Related Indices for Hyperuricemia

**Figure 2:**
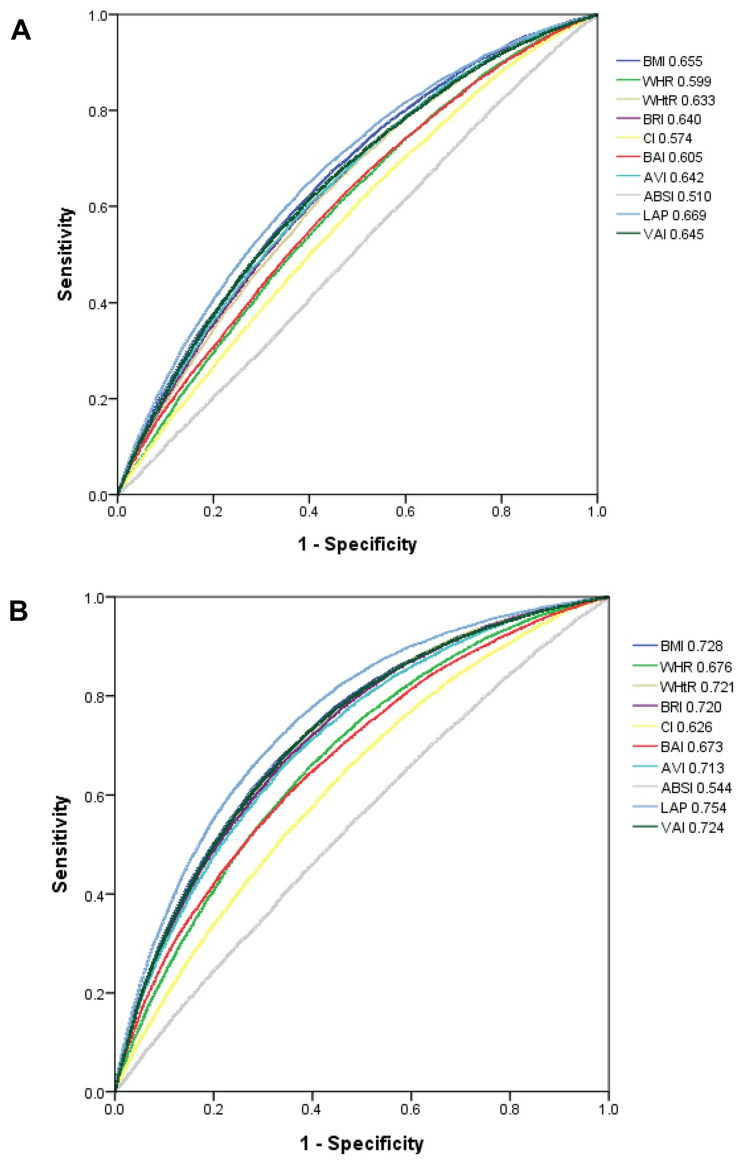
Comparisons of the predictive ability of 10 obesity-related indexes for hyperuricemia among (**A**) men and (**B**) women. Abbreviations: BMI, body mass index; WHR, waist–hip ratio; WHtR, waist-to-height ratio; BRI, body roundness index; CI, conicity index; BAI, body adiposity index; AVI, abdominal volume index; ABSI, a body shape index; LAP, lipid accumulation product; VAI, visceral adiposity index.

The performance and predictive ability of our statistical models to predict the occurrence of hyperuricemia by sex were assessed using ROC curves and AUCs, respectively (Figure 2). In men, LAP had the highest AUC (0.669), followed by BMI (0.655), VAI (0.645), AVI (0.642), BRI (0.640), WHtR (0.633), BAI (0.605), WHR (0.599), CI (0.574), and ABSI (0.510) (Table 3). In women, LAP also had the highest AUC (0.754), followed by BMI (0.728), VAI (0.724), WHtR (0.721), BRI (0.720), AVI (0.713), WHR (0.676), BAI (0.673), CI (0.626), and ABSI (0.544) (Table 3).

We further performed the analysis in all study participants. The Supplemental Table shows the clinical characteristics of the study participants classified by the presence of hyperuricemia (Table S1), the association of obesity-related indices with hyperuricemia using multivariable logistic regression analysis (Table S2), and the AUC of obesity-related indices for hyperuricemia (Table S3) in all study participants.

## 4. Discussion

Our results of sex differences in the relationships among 10 indices of obesity and hyperuricemia showed that all indexes were associated with hyperuricemia in both male and female participants. Moreover, we identified sex differences in the relationship between all 10 indices and hyperuricemia, with stronger associations in women than in men.

We also found that the strongest predictors of hyperuricemia were LAP, BMI, and VAI in both men and women. Many epidemiologic studies have demonstrated a relationship between hyperuricemia with obesity and have also shown that central obesity is a major factor leading to hyperuricemia [43,44]. Central obesity can be evaluated using various indices, and LAP was the strongest predictor for the occurrence of hyperuricemia in both sexes in our results. A Chinese study of 3645 subjects showed that LAP was positively associated with UA in both sexes, and high LAP was significantly associated with an increased OR for hyperuricemia (2.07, 95% confidence interval: 1.66–2.58, *p* < 0.001) [45]. Various biochemical metabolic processes may explain why LAP is a causative factor for the development of hyperuricemia. In the calculation of LAP, there are two important components, WC and TG levels, which are closely related to abdominal fat and lipid metabolism [46]. First, elevated TG levels may result in the overproduction of UA through the classic free fatty acid metabolic pathway [47,48]. Hypertriglyceridemia leads to the overproduction of free fatty acids, and their synthesis in the liver results in the production of purine and, subsequently, the production of UA [49]. Second, obesity is independently associated with low eGFR and increased risk of renal dysfunction, which may decrease urate excretion [50,51]. Lastly, LAP is a risk factor for insulin resistance and has also been associated with the development of hyperuricemia [52,53]. Insulin resistance increases the absorption of urate in the kidneys through the stimulation of urate 1 transport on the membranes of proximal renal tubule brush borders [54,55]. Increased urinary glucose excretion in newly diagnosed patients with diabetes has been shown to increase UA excretion and decrease UA levels [56]. In addition, insulin resistance can increase lipolysis and decrease lipoprotein synthesis, leading to elevated TGs and subsequent increased UA production [13].

BMI was also a strong risk factor for hyperuricemia in this study. Compared to LAP, BMI is calculated using only BW and BH. You A. et al. evaluated 4360 participants from the Shanghai Elderly Cardiovascular Health study cohort in 2017 and found high BMI was associated with hyperuricemia [37]. In addition, Li S. et al. investigated 19,343 Chinese adults and found serum UA was positively associated with BMI [38]. Studies on Chinese, Japanese, and American patients have all shown a linear dose–response relationship between BMI and hyperuricemia. With an increase in BMI, the OR of hyperuricemia increased. For lean patients in Japan (BMI < 18.5 kg/m^2^), the OR for hyperuricemia was 0.514 compared with 1.867, 2.929, and 4.738 in overweight (25 ≤ BMI < 30 kg/m^2^), obese (30 ≤ BMI < 35 kg/m^2^), and severely obese (35 kg/m^2^ ≤ BMI) patients, respectively. For overweight (25 ≤ BMI < 30 kg/m^2^), obese (30 ≤ BMI < 35 kg/m^2^), and severely obese (35 kg/m^2^ ≤ BMI) patients in the United States, the ORs for hyperuricemia were 2.027, 3.196, and 5.099, respectively [57,58]. Another retrospective study focusing on healthy subjects in the Jiangsu province of China indicated a positive relationship between BMI and serum UA levels [36]. Several possible biochemical mechanisms could explain this relationship. Hyperuricemia is caused by high levels of UA, which are strongly influenced by the overproduction and decrease in the excretion and clearance of UA [47]. Furthermore, the accumulation of visceral fat causes a large increase in plasma free fatty acids in the liver and hepatic portal veins, thereby promoting TG synthesis and, subsequently, UA synthesis, resulting in increased UA levels [47,59]. Therefore, higher BMI in patients with more fat accumulation may increase the likelihood of developing hyperuricemia. However, BMI only involves BW and BH and should therefore be a weaker risk factor for hyperuricemia than LAP, which is calculated using serum TG levels. VAI was also a strong risk factor for hyperuricemia in the present study. Tian X. et al. evaluated 56,537 participants without hyperuricemia and underwent two health examinations during 2006–2008 from the Kailuan study [60]. They found cumulative abnormal VAI burdens were positively associated with the risk of hyperuricemia, especially in females. VAI has been reported to be a strong and independent indicator of hyperuricemia among individuals without metabolic syndrome (OR, 3.077; 95% confidence interval, 1.789–5.293; *p* < 0.001) [61]. The mechanisms linking visceral fat accumulation with hyperuricemia include excessive production and reduced renal excretion of UA [62,63]. Many studies have suggested the metabolically active nature of visceral adipose tissue and that this can lead to the dysregulation of adipocytokines and, subsequently, hyperinsulinemia [64,65,66]. Hyperinsulinemia has been associated with increases in sodium and UA reabsorption, which affects renal ducts and reduces urinary and sodium excretion, leading to increased hyperuricemia [67,68]. In summary, LAP was the strongest risk factor for hyperuricemia in both men and women in this study, followed by BMI and VAI. Further studies are needed to clarify the underlying mechanisms.

Another important finding of this study is that all 10 of the studied indices were more closely related to hyperuricemia in women than in men. Many previous studies have shown sex differences in levels of plasma xanthine oxidoreductase (XOR), hypoxanthine, and xanthine. XOR catabolizes the reaction from hypoxanthine to xanthine and further to UA [69]. Using a mouse model, Tsushima et al. demonstrated that adipose tissue itself is rich in xanthine oxidoreductase, which can lead to the production of UA and further promote obesity [70]. Many previous studies have reported significantly higher levels of xanthine, hypoxanthine, and plasma XOR in men than in women and that the concentration of hypoxanthine, but not that of xanthine, has been independently associated with obesity [71]. Another study reported that exercise resulted in a marked elevation in the level of serum hypoxanthine in obese subjects compared with control subjects [72]. In the present study, we found a stronger relationship between the obesity indices and hyperuricemia in women than in men. We hypothesize that this may be due to greater XOR activity in men and, consequently, increases in the reaction from hypoxanthine to xanthine compared to women. Since the concentration of hypoxanthine is independently associated with obesity, the higher XOR activity in men may lead to a weaker association between obesity indices and hyperuricemia. A study on sex differences in metabolic syndrome also showed that women with general obesity had a higher risk of significant hyperuricemia compared to men [73]. In summary, stronger associations were found between each obesity index and hyperuricemia in the women in this study. Further research into the sexual physiology associated with hyperuricemia and obesity is needed.

The main strengths of this study include the comprehensive assessments of 10 obesity indices and their associations with hyperuricemia in a large cohort of community-dwelling men and women. Several limitations should also be noted. First, we lacked data on prescriptions for urate- and lipid-lowering drugs or for drugs used to treat diabetes and hypertension, as this information is not recorded in the Taiwan Biobank. These drugs could have influenced the occurrence of hyperuricemia and, consequently, led to underestimation of the associations. Second, we did not evaluate the duration of hyperuricemia in this cross-sectional study, and, consequently, analysis of causal relationships was not possible. Further longitudinal studies are warranted to clarify our results. Third, we had no data on physical activity and dietary habits, which might have affected the serum UA value. Fourth, several genetic polymorphisms of xanthine oxidoreductase had been identified, but we had no genetic data to assess the relationship between hyperuricemia and genetic variations. Finally, as the Taiwan Biobank only recruits enrollees of Chinese ethnicity, the conclusions may not be applicable to other areas or ethnic groups.

In conclusion, we identified associations between all 10 studied obesity indices (BMI, WHR, WHtR, BRI, CI, BAI, AVI, ABSI, LAP, and VAI) and hyperuricemia in a large community-dwelling cohort of Taiwanese adults. We also identified sex differences in these associations and suggested possible mechanisms.

## Figures and Tables

**Table 1:** Differences between the male and female participants with and without hyperuricemia in clinical characteristics.

Characteristic		Man (*n* = 43,790)		Woman (*n* = 78,098)		
	Hyperuricemia (−) (*n* =30,735)	Hyperuricemia (+) (*n* = 13,055)	*p*	Hyperuricemia (−) (*n* = 67,494)	Hyperuricemia (+) (*n* = 10,604)	*p*
Age (year)	50.4 ± 11.3	48.6 ± 11.4	<0.001	49.3 ± 10.7	53.4 ± 10.3	<0.001
DM (%)	7.5	5.2	<0.001	3.7	7.8	<0.001
Hypertension (%)	15.5	20.0	<0.001	7.7	22.0	<0.001
Smoking history (%)	56.6	59.5	<0.001	10.3	10.7	0.291
Systolic BP (mmHg)	124.5 ± 16.5	127.5 ± 17.0	<0.001	115.1 ± 17.6	124.4 ± 19.0	<0.001
Diastolic BP (mmHg)	77.2 ± 10.5	80.1 ± 11.0	<0.001	70.1 ± 10.2	75.1 ± 11.0	<0.001
Body height (cm)	169.5 ± 6.3	170.0 ± 6.3	<0.001	157.7 ± 5.7	156.7 ± 5.6	<0.001
Body weight (kg)	71.4 ± 11.1	77.2 ± 12.4	<0.001	57.6 ± 9.3	64.7 ± 11.6	<0.001
Waist circumference (cm)	86.6 ± 9.1	91.2 ± 9.3	<0.001	79.7 ± 9.3	87.2 ± 10.4	<0.001
Hip circumference (cm)	96.7 ± 6.4	99.7 ± 7.0	<0.001	94.6 ± 6.8	98.7 ± 8.5	<0.001
Laboratory parameters						
Uric acid (mg/dL)	5.7 ± 0.9	8.0 ± 0.9	<0.001	4.6 ± 0.8	6.8 ± 0.8	<0.001
Fasting glucose (mg/dL)	99.8 ± 25.4	98.2 ± 17.6	<0.001	93.2 ± 18.4	98.7 ± 20.3	<0.001
Hemoglobin (g/dL)	15.0 ± 1.2	15.2 ± 1.2	<0.001	13.0 ± 1.3	13.4 ± 1.2	<0.001
Triglyceride (mg/dL)	125.1 ± 102.8	167.7 ± 142.8	<0.001	96.4 ± 66.5	145.6 ± 103.7	<0.001
Total cholesterol (mg/dL)	189.7 ± 34.4	197.1 ± 36.2	<0.001	196.2 ± 35.5	207.3 ± 38.3	<0.001
HDL-c (mg/dL)	49.1 ± 11.3	45.4 ± 10.3	<0.001	59.2 ± 13.2	52.1 ± 11.9	<0.001
LDL-c (mg/dL)	120.2 ± 31.0	125.3 ± 32.3	<0.001	119.0 ± 31.3	130.3 ± 33.9	<0.001
eGFR (mL/min/1.73 m^2^)	102.4 ± 20.3	93.1 ± 20.1	<0.001	117.7 ± 25.5	100.8 ± 24.6	<0.001
Obesity-related indices						
BMI (kg/m^2^)	24.8 ± 3.4	26.7 ± 3.6	<0.001	23.2 ± 3.5	26.3 ± 4.3	<0.001
WHR (%)	89.5 ± 5.6	91.4 ± 5.4	<0.001	84.1 ± 6.7	88.3 ± 6.6	<0.001
WHtR (%)	51.1 ± 5.4	53.7 ± 5.4	<0.001	50.6 ± 6.1	55.7 ± 6.8	<0.001
BRI	7.0 ± 1.7	7.8 ± 1.9	<0.001	6.3 ± 1.8	7.8 ± 2.2	<0.001
CI	1.23 ± 0.07	1.24 ± 0.06	<0.001	1.21 ± 0.08	1.25 ± 0.09	<0.001
BAI	25.9 ± 3.1	27.0 ± 3.2	<0.001	29.8 ± 3.8	32.4 ± 4.5	<0.001
AVI	15.3 ± 3.2	16.9 ± 3.5	<0.001	13.0 ± 3.0	15.6 ± 3.8	<0.001
ABSI	0.0784 ± 0.0038	0.0785 ± 0.0037	<0.001	0.0783 ± 0.0052	0.0791 ± 0.0052	<0.001
LAP	33.2 ± 37.2	51.9 ± 49.7	<0.001	25.5 ± 25.1	49.5 ± 40.7	<0.001
VAI	1.6 ± 1.9	2.3 ± 2.7	<0.001	1.5 ± 1.6	2.6 ± 2.5	<0.001

Abbreviations: DM, diabetes mellitus; BP, blood pressure; HDL-c, high-density lipoprotein cholesterol; LDL-c, low-density lipoprotein cholesterol; eGFR, estimated glomerular filtration rate; BMI, body mass index; WHR, waist–hip ratio; WHtR, waist-to-height ratio; BRI, body roundness index; CI, conicity index; BAI, body adiposity index; AVI, abdominal volume index; ABSI, a body shape index; LAP, lipid accumulation product; VAI, visceral adiposity index.

**Table 2:** Associations between the obesity-related indexes and hyperuricemia in multivariable logistic regression analysis by sex.

Obesity-Related Index	Man (*n* = 43,790)			Woman (*n* = 78,098)			β	Interaction *p*
	Multivariable			Multivariable				
	Odds Ratio	95% Confidence Interval	*p*	Odds Ratio	95% Confidence Interval	*p*		
BMI (per 1 kg/m^2^) ^a^	1.121	1.113–1.129	<0.001	1.163	1.155–1.170	<0.001	0.058	<0.001
WHR (per 0.01) ^a^	1.058	1.053–1.063	<0.001	1.054	1.050–1.058	<0.001	0.036	<0.001
WHtR (per 0.01) ^a^	1.077	1.072–1.082	<0.001	1.090	1.085–1.094	<0.001	0.043	<0.001
BRI (per 1) ^a^	1.241	1.224–1.258	<0.001	1.296	1.281–1.312	<0.001	0.128	<0.001
CI (per 0.1) ^a^	1.460	1.406–1.516	<0.001	1.340	1.303–1.378	<0.001	0.196	<0.001
BAI (per 1) ^a^	1.095	1.087–1.103	<0.001	1.113	1.107–1.120	<0.001	0.048	<0.001
AVI (per 1) ^a^	1.119	1.111–1.128	<0.001	1.162	1.154–1.170	<0.001	0.077	<0.001
ABSI (per 0.01) ^a^	1.260	1.183–1.341	<0.001	1.088	1.040–1.138	<0.001	0.244	<0.001
LAP (per 1) ^b^	1.010	1.009–1.011	<0.001	1.018	1.017–1.019	<0.001	0.009	<0.001
VAI (per 1) ^c^	1.238	1.219–1.258	<0.001	1.339	1.321–1.357	<0.001	0.078	<0.001

Abbreviations: BMI, body mass index; WHR, waist–hip ratio; WHtR, waist-to-height ratio; BRI, body roundness index; CI, conicity index; BAI, body adiposity index; AVI, abdominal volume index; ABSI, a body shape index; LAP, lipid accumulation product; VAI, visceral adiposity index. ^a^ Covariates in the multivariable model included age, DM, hypertension, smoking history, systolic and diastolic BPs, hemoglobin, triglyceride, total cholesterol, HDL-c, LDL-c, and eGFR. ^b^ Covariates as ^a^ covariates, except for triglycerides. ^c^ Covariates as ^a^ covariates, except for triglycerides and HDL-c.

**Table 3:** Area under curve of obesity-related indexes for hyperuricemia by sex.

Obesity-Related Index	Man (*n* = 43,790)			Woman (*n* = 78,098)			Interaction *p*
	AUC	95% Confidence Interval	*p*	AUC	95% Confidence Interval	*p*	
BMI (per 1 kg/m^2^)	0.655	0.649–0.660	<0.001	0.728	0.723–0.733	<0.001	<0.001
WHR (per 0.01)	0.599	0.594–0.605	<0.001	0.676	0.671–0.982	<0.001	<0.001
WHtR (per 0.01)	0.633	0.628–0.639	<0.001	0.721	0.716–0.726	<0.001	<0.001
BRI (per 1)	0.640	0.634–0.645	<0.001	0.720	0.715–0.725	<0.001	<0.001
CI (per 0.1)	0.574	0.568–0.579	<0.001	0.626	0.620–0.631	<0.001	<0.001
BAI (per 1)	0.605	0.600–0.611	<0.001	0.673	0.668–0.679	<0.001	<0.001
AVI (per 1)	0.642	0.637–0.648	<0.001	0.713	0.708–0.718	<0.001	<0.001
ABSI (per 0.01)	0.510	0.504–0.516	0.001	0.544	0.538–0.550	<0.001	<0.001
LAP (per 1)	0.669	0.664–0.675	<0.001	0.754	0.750–0.759	<0.001	<0.001
VAI (per 1)	0.645	0.640–0.651	<0.001	0.724	0.719–0.729	<0.001	<0.001

Abbreviations: AUC, area under the curve; BMI, body mass index; WHR, waist–hip ratio; WHtR, waist-to-height ratio; BRI, body roundness index; CI, conicity index; BAI, body adiposity index; AVI, abdominal volume index; ABSI, a body shape index; LAP, lipid accumulation product; VAI, visceral adiposity index.

## Data Availability

The data underlying this study are from the Taiwan Biobank. Due to restrictions placed on the data by the Personal Information Protection Act of Taiwan, the minimal data set cannot be made publicly available. Data may be available upon request to interested researchers. Please send data requests to: Szu-Chia Chen. Division of Nephrology, Department of Internal Medicine, Kaohsiung Medical University Hospital, Kaohsiung Medical University.

## Supplementary Materials

The following supporting information can be downloaded at: https://www.mdpi.com/article/10.3390/nu15153419/s1, Table S1: clinical characteristics of the study participants classified by the presence of hyperuricemia, Table S2: association of obesity-related indices with hyperuricemia using multivariable logistic regression analysis, Table S3: area under curve of obesity-related indices for hyperuricemia.
