# Supplementary material for: Sex Difference in the Associations among Obesity-Related Indices with Hyperuricemia in a Large Taiwanese Population Study
Source: Nutrients. 2023 Aug 1;15(15):3419. doi: 10.3390/nu15153419 (PMC10421218; doi:10.3390/nu15153419)
Supplement: Supplementary file 1 [file nutrients-15-03419-s001.zip › nutrients-2532780-supplementary.pdf]

Table S1. Clinical characteristics of the study participants classified by the presence of hyperuricemia.

Characteristics	Hyperuricemia (−) (n = 98,229)	Hyperuricemia (+) (n = 23,659)	p
Age (year)	49.7 ± 10.9	50.8 ± 11.2	<0.001
Sex (men) (%)	31.3	55.2	<0.001
DM (%)	4.9	6.3	<0.001
Hypertension (%)	10.2	20.9	<0.001
Smoking history (%)	24.8	37.6	<0.001
Systolic BP (mmHg)	118.1 ± 17.8	126.1 ± 18.0	<0.001
Diastolic BP (mmHg)	72.3 ± 10.8	77.9 ± 11.2	<0.001
Body height (cm)	161.4 ± 8.0	164.0 ± 8.9	<0.001
Body weight (kg)	61.9 ± 11.8	71.6 ± 13.6	<0.001
Waist circumference (cm)	81.8 ± 9.7	89.4 ± 10.0	<0.001
Hip circumference (cm)	95.2 ± 6.8	99.2 ± 7.7	<0.001
Laboratory parameters			<0.001
Uric acid (mg/dL)	4.9 ± 1.0	7.5 ± 1.0	<0.001
Fasting glucose (mg/dL)	95.3 ± 21.1	98.5 ± 18.8	<0.001
Hemoglobin (g/dL)	13.6 ± 1.6	14.4 ± 1.5	<0.001
Triglyceride (mg/dL)	105.4 ± 80.7	157.8 ± 127.3	<0.001
Total cholesterol (mg/dL)	194.2 ± 35.3	201.7 ± 37.5	<0.001
HDL-c (mg/dL)	56.0 ± 13.5	48.4 ± 11.6	<0.001
LDL-c (mg/dL)	119.3 ± 31.2	127.5 ± 33.1	<0.001
eGFR (mL/min/1.73 m^2)	112.9 ± 25.0	96.5 ± 22.5	<0.001
Obesity-related indices			<0.001
BMI (kg/m^2)	23.7 ± 2.5	26.5 ± 4.0	<0.001
WHR (%)	85.8 ± 6.8	90.0 ± 6.2	<0.001
WHtR (%)	50.7 ± 5.9	54.6 ± 6.1	<0.001
BRI	6.5 ± 1.8	7.8 ± 2.0	<0.001
CI	1.22 ± 0.08	1.25 ± 0.07	<0.001
BAI	28.6 ± 4.0	29.4 ± 4.7	<0.001
AVI	13.7 ± 3.2	16.3 ± 3.7	<0.001
ABSI	0.0783 ± 0.0048	0.0788 ± 0.0045	<0.001
LAP	27.9 ± 29.6	50.8 ± 45.9	<0.001
VAI	1.5 ± 1.7	2.5 ± 2.6	<0.001

Abbreviations: DM, diabetes mellitus; BP, blood pressure; HDL-c, high-density lipoprotein cholesterol; LDL-c, low-density lipoprotein cholesterol; eGFR, estimated glomerular filtration rate; BMI, body mass index; WHR, waist–hip ratio; WHtR, waist-to-height ratio; BRI, body roundness index; CI, conicity index; BAI, body adiposity index; AVI, abdominal volume index; ABSI, a body shape index; LAP, lipid accumulation product; VAI, visceral adiposity index.

Table S2. Association of obesity-related indices with hyperuricemia using multivariable logistic regression analysis.

Obesity-Related Indices	OR	95% Confidence Interval	*p*
BMI (per 1 kg/m^2) [a]	1.143	1.138-1.148	<0.001
WHR (per 0.01) [a]	1.057	1.054-1.060	<0.001
WHtR (per 0.01) [a]	1.085	1.081-1.088	<0.001
BRI (per 1) [a]	1.272	1.261-1.284	<0.001
CI (per 0.1) [a]	1.389	1.358-1.420	<0.001
BAI (per 1) [a]	1.106	1.101-1.111	<0.001
AVI (per 1) [a]	1.140	1.135-1.146	<0.001
ABSI (per 0.01) [a]	1.156	1.115-1.198	<0.001
LAP (per 1) [b]	1.013	1.012-1.014	<0.001
VAI (per 1) [c]	1.306	1.293-1.319	<0.001

Values expressed as odds ratio (OR) and 95% confidence interval. Abbreviations: BMI, body mass index; WHR, waist–hip ratio; WHtR, waist-to-height ratio; BRI, body roundness index; CI, conicity index; BAI, body adiposity index; AVI, abdominal volume index; ABSI, a body shape index; LAP, lipid accumulation product; VAI, visceral adiposity index.

[a] Covariates in the multivariable model included age, DM, hypertension, smoking history, systolic and diastolic BPs, hemoglobin, triglyceride, total cholesterol, HDL-c, LDL-c and eGFR.

[b] Covariates as [a]Covariates, except for triglyceride.

[c] Covariates as [a]Covariates, except for triglyceride and HDL-c.

Table S3. Area under curve of obesity-related indices for hyperuricemia.

Obesity-Related Indices	AUC	95% Confidence Interval	*p*
BMI (per 1 kg/m^2) [a]	0.716	0.713-0.720	<0.001
WHR (per 0.01) [a]	0.680	0.676-0.683	<0.001
WHtR (per 0.01) [a]	0.680	0.677-0.684	<0.001
BRI (per 1) [a]	0.702	0.699-0.706	<0.001
CI (per 0.1) [a]	0.612	0.608-0.615	<0.001
BAI (per 1) [a]	0.543	0.539-0.547	<0.001
AVI (per 1) [a]	0.712	0.709-0.716	<0.001
ABSI (per 0.01) [a]	0.529	0.525-0.533	<0.001
LAP (per 1) [b]	0.725	0.722-0.729	<0.001
VAI (per 1) [c]	0.686	0.682-0.690	<0.001

Values expressed as area under curve (AUC) and 95% confidence interval. Abbreviations: BMI, body mass index; WHR, waist–hip ratio; WHtR, waist-to-height ratio; BRI, body roundness index; CI, conicity index; BAI, body adiposity index; AVI, abdominal volume index; ABSI, a body shape index; LAP, lipid accumulation product; VAI, visceral adiposity index.

[a] Covariates in the multivariable model included age, DM, hypertension, smoking history, systolic and diastolic BPs, hemoglobin, triglyceride, total cholesterol, HDL-c, LDL-c and eGFR.

[b] Covariates as [a]Covariates, except for triglyceride.

[c] Covariates as [a]Covariates, except for triglyceride and HDL-c.
